# Influenza virus-related critical illness: prevention, diagnosis, treatment

**DOI:** 10.1186/s13054-019-2491-9

**Published:** 2019-06-12

**Authors:** Eric J. Chow, Joshua D. Doyle, Timothy M. Uyeki

**Affiliations:** 1 Epidemic Intelligence Service, Centers for Disease Control and Prevention, Atlanta, GA USA; 2 Influenza Division, National Center for Immunization and Respiratory Diseases, Centers for Disease Control and Prevention, Mailstop H24-7, 1600 Clifton Road, N.E., Atlanta, GA 30329 USA

**Keywords:** Influenza, Influenza vaccination, Influenza testing, Antiviral treatment

## Abstract

Annual seasonal influenza epidemics of variable severity result in significant morbidity and mortality in the United States (U.S.) and worldwide. In temperate climate countries, including the U.S., influenza activity peaks during the winter months. Annual influenza vaccination is recommended for all persons in the U.S. aged 6 months and older, and among those at increased risk for influenza-related complications in other parts of the world (e.g. young children, elderly). Observational studies have reported effectiveness of influenza vaccination to reduce the risks of severe disease requiring hospitalization, intensive care unit admission, and death. A diagnosis of influenza should be considered in critically ill patients admitted with complications such as exacerbation of underlying chronic comorbidities, community-acquired pneumonia, and respiratory failure during influenza season. Molecular tests are recommended for influenza testing of respiratory specimens in hospitalized patients. Antigen detection assays are not recommended in critically ill patients because of lower sensitivity; negative results of these tests should not be used to make clinical decisions, and respiratory specimens should be tested for influenza by molecular assays. Because critically ill patients with lower respiratory tract disease may have cleared influenza virus in the upper respiratory tract, but have prolonged influenza viral replication in the lower respiratory tract, an endotracheal aspirate (preferentially) or bronchoalveolar lavage fluid specimen (if collected for other diagnostic purposes) should be tested by molecular assay for detection of influenza viruses.

Observational studies have reported that antiviral treatment of critically ill adult influenza patients with a neuraminidase inhibitor is associated with survival benefit. Since earlier initiation of antiviral treatment is associated with the greatest clinical benefit, standard-dose oseltamivir (75 mg twice daily in adults) for enteric administration is recommended as soon as possible as it is well absorbed in critically ill patients. Based upon observational data that suggest harms, adjunctive corticosteroid treatment is currently not recommended for children or adults hospitalized with influenza, including critically ill patients, unless clinically indicated for another reason, such as treatment of asthma or COPD exacerbation, or septic shock. A number of pharmaceutical agents are in development for treatment of severe influenza.

## Background

Annual seasonal influenza epidemics of variable severity result in significant morbidity and mortality in the United States (U.S.) and worldwide [1–3]. In temperate climate countries, including the U.S., influenza activity peaks during the winter months whereas in tropical regions influenza activity may be more variable [4–6]. Most persons with symptomatic influenza virus infection have self-limited uncomplicated upper respiratory tract illness. One study estimated that during 2010–2016, approximately 8.3% of the U.S. population experienced symptomatic influenza each year [7]. However, complications may result in severe illness, including fatal outcomes. During 2010–2018, an estimated 4.3–23 million medical visits, 140,000–960,000 hospitalizations, and 12,000–79,000 deaths were associated with influenza each year in the U.S. [8]. Another study estimated that 18,000–96,000 influenza-related intensive care unit (ICU) admissions occur annually in the U.S. [9]. There are an estimated 291,000–646,000 respiratory deaths attributed to seasonal influenza each year worldwide [2]. Here, we review strategies for prevention, diagnosis, and treatment of influenza virus infections in the ICU (Table 1).

**Table 1:** Key points: care of patients with severe influenza

Key Points	
• There are an estimated 291,000–646,000 seasonal influenza-associated respiratory deaths every year worldwide.	
• Annual influenza vaccination is the primary method of preventing influenza and influenza-related complications, especially in high-risk persons.	
• Influenza molecular diagnostic testing is recommended for all patients requiring hospitalization with suspected influenza.	
• Influenza antiviral treatment should be started as soon as possible in hospitalized patients with suspected influenza, including critically ill patients, and should not be delayed while awaiting results of influenza diagnostic tests.	
• Enterically administered oseltamivir is recommended for influenza patients except for those with contraindications (e.g., gastric stasis, ileus, malabsorption).	
• Repeat virologic testing in lower respiratory tract specimens may be required to determine therapeutic endpoints in ventilated patients with influenza	
• Corticosteroids are not recommended for the routine treatment of influenza except when indicated for treatment of underlying medical conditions (e.g., COPD or asthma exacerbation) or septic shock.	

## Risk factors

Influenza vaccination is the primary method for preventing influenza and reducing the risk of severe outcomes. In the U.S., the Advisory Committee on Immunization Practices (ACIP) recommends annual influenza vaccination for all persons aged 6 months and older and prioritizes those at higher risk for influenza complications [10]. High-risk groups include adults aged > 65 years [11, 12], children aged < 5 years (particularly those aged < 2 years) [13, 14], pregnant women (up to 2 weeks post-partum) [15–18], persons with certain chronic medical conditions, Native Americans/Alaska Natives,^1^ and residents of nursing homes and other long-term care facilities (Table 2). Studies have specifically highlighted that those with chronic pulmonary, cardiovascular, renal, hepatic, neurologic, hematologic or metabolic disorders, immunocompromised persons, children and adolescents receiving aspirin- or salicylate-containing medications and who might be at risk for experiencing Reye syndrome with influenza virus infection, and those who are extremely obese (BMI > 40) are at increased risk for influenza-related complications [10, 19–23].

**Table 2:** Groups at high risk for influenza complications*

Risk factors for severe influenza outcomes	
• Age < 5 years, especially those < 2 years	
• Age > 65 years	
• Pregnant women	
• Extreme obesity (BMI > 40 kg/m^2^)	
• Native Americans/Alaskan Natives (may also apply to indigenous people from other countries)	
• Current or past tobacco use	
• Children and adolescents receiving aspirin or salicylate-containing medications who might be at risk for Reye syndrome	
• Underlying chronic medical conditions:	
○ Pulmonary	
○ Cardiovascular	
○ Renal	
○ Hepatic	
○ Neurologic	
○ Hematologic	
○ Metabolic	
○ Immunocompromised state	

*From the U.S. Centers for Disease Control and Prevention Advisory Committee on Immunization Practices

Many studies evaluated risk factors for severe influenza during the 2009 H1N1 influenza pandemic. Adult ICU patients with influenza A(H1N1)pdm09 virus infection were primarily non-elderly, were obese [24–28], and had higher odds of death, invasive mechanical ventilation, acute respiratory distress syndrome (ARDS), septic shock, and multi-lobar pneumonia when compared with seasonal influenza patients [24, 29]. In children, independent risk factors for influenza A(H1N1)pdm09-related mortality included chronic neurologic condition or immune compromise, acute myocarditis or encephalitis, and early presumed MRSA co-infection of the lung [30]. Female gender was also identified as a risk factor; however, there was no gender difference in overall mortality. Bacterial co-infection was identified in approximately one third of fatal influenza A(H1N1)pdm09 cases in the largest autopsy case series [31]. Bacterial co-infections in the inter-pandemic period are also common in critically ill influenza patients [32]. One study identified past or current tobacco use as a risk factor associated with ICU admission [33]. A recent multicenter cohort study reported that mortality was higher in immunosuppressed patients with influenza A(H1N1)pdm09 than in immunocompetent patients [34]. Severity of influenza seasons varies from year-to-year based on the predominant influenza viruses, and between seasonal and pandemic influenza [35, 36]. One study reported that patients with influenza A(H1N1)pdm09 had higher odds of severe disease than patients with either influenza A(H3N2) or influenza B virus infections [37]. However, influenza B virus infection has been shown to increase the odds of in-hospital mortality in children compared with influenza A virus infection [38].

## Prevention and vaccination

Influenza vaccination is recommended each fall for all persons aged > 6 months in the U.S. and should continue as long as influenza viruses are circulating in the community. Previously unvaccinated children aged 6 months through 8 years require two doses 1 month apart. Since influenza vaccine effectiveness (VE) to prevent medically attended illness varies from year-to-year by vaccine strain, age, prior immunity, and immune function, some vaccinated individuals can become symptomatic with influenza virus infection. However, several studies have reported influenza vaccine effectiveness in reducing illness severity, including reducing severe illness in persons aged > 65 years [39], and reducing in-hospital mortality and ICU admissions for those aged 18–49 years and > 65 years compared to unvaccinated individuals [40]. One study reported that duration of ICU hospitalization was reduced a half-day in patients aged 50–64 years who had received influenza vaccination compared with unvaccinated patients [41]. A study across all age groups in Spain reported influenza VE of 58% in reducing the risk of severe influenza requiring hospitalization [42]. A Southern Hemisphere study reported influenza VE of 82% in reducing influenza-associated ICU admissions among adults [43] while a study in Spain showed an adjusted influenza VE of 23% in preventing ICU admission and death [44].

Despite the benefits of influenza vaccination, there continues to be low vaccine coverage among adults admitted to the ICU who often have a high prevalence of high-risk comorbidities [45, 46]. In children, low influenza vaccination coverage has also been reported among those admitted to pediatric ICUs, even among those with underlying high-risk conditions [47]. Full influenza vaccination was shown to result in a 74% reduction in pediatric ICU admissions compared to unvaccinated or partially vaccinated influenza patients [47]. Furthermore, one study showed that influenza VE was 65% in reducing the risk of mortality in children aged 6 months to 17 years in the U.S. [48]. These data further emphasize the benefits of influenza vaccination in reducing severe influenza complications, especially in high-risk persons.

## Diagnosis

Persons with uncomplicated influenza typically experience acute onset of respiratory symptoms (cough, rhinorrhea, congestion), myalgias, and headache with or without fever. During influenza season, clinicians should also consider influenza when there is only fever present or in patients who are afebrile and have respiratory symptoms [49]. Complications of influenza vary by age, underlying comorbidities or high-risk conditions such as pregnancy, and immune function; elderly and immunocompromised persons may not always manifest fever. Critically ill patients may be admitted with respiratory or multi-organ failure, exacerbation of an underlying condition such as chronic lung disease [50, 51], heart failure [52], or other extrapulmonary complications including stroke, encephalopathy, or encephalitis [30, 49, 53].

Influenza testing is recommended for all patients requiring hospitalization with suspected influenza, including those admitted to the ICU during influenza season with acute respiratory illness and community-acquired pneumonia, without a clear alternative diagnosis. Furthermore, all individuals requiring critical care outside of influenza season should be tested for influenza if there is a possible epidemiological link to an individual with recent influenza, such as travel to areas with influenza activity or exposure to an institutional influenza outbreak. Special consideration should be given to elderly and immunocompromised patients, as influenza virus infection may not present with typical acute respiratory illness signs and symptoms (e.g., absence of fever). The Infectious Diseases Society of America (IDSA) 2018 Influenza Clinical Practice Guidelines also recommend influenza testing for patients at high risk of complications such as exacerbation of chronic cardiopulmonary disease [49]. Diagnosis of influenza should be made as soon as possible in critically ill patients, and initiation of antiviral treatment should not be delayed while awaiting results of diagnostic tests. Studies have reported an increase in mortality of ICU patients with influenza A(H1N1)pdm09 virus infection when diagnosis was delayed [54], and shorter hospital length of stay when antiviral treatment was initiated within 6 h of admission [55].

Several kinds of influenza diagnostic tests are available in clinical settings with variable sensitivities and specificities, including antigen detection assays, and molecular assays (nucleic acid detection) using respiratory tract specimens (Table 3). Within each of these testing categories, there is a wide range of available tests with varying diagnostic accuracy, and understanding the limitations of each diagnostic tool will allow the clinician to properly interpret their results. Most studies of influenza diagnostic accuracy have been conducted on specimens from patients with uncomplicated influenza, and few have assessed the performance of influenza tests in critically ill patients. The IDSA guidelines recommend molecular influenza assays for testing respiratory specimens from all hospitalized patients with suspected influenza because of their high sensitivity, specificity, and time to results (15 min to several hours) [49]. The use of rapid influenza molecular diagnostic testing can result in better outcomes for patients and reduce the amount of resources required to care for patients in the emergency room [57]. Serology and viral culture are not recommended for clinical decision making, because timely results will not be available to inform clinical management. Serology requires collection of appropriately paired acute and convalescent sera performed at specialized public health reference laboratories, and results based upon a single serum specimen are not interpretable [49]. Although viral culture can confirm the presence of infectious virus with very high sensitivity and specificity, it must be performed at public health laboratories and requires 3–10 days to yield results.

**Table 3:** Influenza diagnostic tests

Influenza testing modality[49, 56]	Method	Time to results	Sensitivity	Specificity	Respiratory specimens*		
					Swab	Wash/fluid	Aspirate
Molecular assay (Rapid)**#	Nucleic acid amplification	10–15 min	Moderate to high	High	NP or nasal	N/A	N/A
Molecular assay**#	Nucleic acid amplification	15–30 min	High	High	NP or throat	NP or BAL/mini BAL	Nasal or endotracheal
Rapid influenza diagnostic Test (RIDT)	Antigen detection	10–15 min	Low to moderate	High	NP, nasal, throat	NP or nasal	NP or nasal
Immunofluorescence assay (direct and indirect)	Antigen detection	1–4 h	Moderate	High	NP	NP	Nasal
Rapid cell culture (shell vials; cell mixtures)	Virus isolation	1–3 days	High	High	NP or throat	NP or BAL/mini BAL	Nasal or endotracheal
Tissue cell viral culture (conventional)	Virus isolation	3–10 days	High	High	NP or throat	NP or BAL/mini BAL	Nasal or endotracheal

*FDA-approved clinical specimens vary by specific test; refer to the manufacturer’s package insert for each test’s approved specimens

**Recommended for testing hospitalized patients with suspected influenza. Some molecular assays also detect other respiratory pathogens

#Patients with respiratory failure and suspected influenza should have lower respiratory tract specimens collected and tested, including if upper respiratory tract specimens are negative for influenza because a patient may have cleared influenza virus from the upper respiratory tract and continue to have influenza viral replication in the lower respiratory tract

*NP* nasopharyngeal, *BAL* bronchoalveolar lavage

Serologic testing is not recommended for diagnosis or clinical management of patients with suspected influenza

A recent meta-analysis reported that influenza antigen detection tests that produce rapid results had very high specificities (> 98%), but sensitivities were highly variable compared with RT-PCR [58]. Rapid influenza diagnostic tests (RIDTs) without an analyzer device had only moderate sensitivity (53–54%), RIDTs that utilize an analyzer device (digital immunoassays) had moderately high sensitivity (77–80%), and rapid influenza molecular assays (nucleic acid detection) had high sensitivity (92–95%) [58]. Low sensitivity of RIDTs for detecting influenza virus in ICU patients has been reported [59]. Recently, a systematic analysis of rapid influenza molecular tests from 29 studies reported pooled sensitivity and specificity of 87.9% and 97.4%, respectively [60]. Therefore, antigen detection assays, such as rapid influenza diagnostic tests and immunofluorescence assays, are not recommended for hospitalized patients with suspected influenza because of their lower sensitivities, unless molecular assays are not available [49]. Negative results for influenza based on tests with low sensitivity (e.g., RIDTs, immunofluorescence assays) should not be used to make clinical decisions. Instead, negative test results should be followed up with reverse transcription polymerase chain reaction (RT-PCR) or other influenza molecular assays to confirm results, and antiviral treatment should continue until results are available.

Preferred respiratory specimens for influenza testing in hospitalized patients without lower respiratory tract disease include nasopharyngeal, mid-turbinate nasal, or combined nasal-throat swabs. Collection of lower respiratory tract specimens should be considered in hospitalized patients with suspected influenza if upper respiratory tract specimens are negative and a positive test would result in a change of clinical management [61], because viral replication in the lower respiratory tract may be ongoing and prolonged after virus is no longer detectable in the upper respiratory tract [24, 25]. Influenza A(H1N1)pdm09 virus in particular has been shown to have affinity for infecting the lower respiratory tract [24, 31]. In hospitalized patients receiving invasive mechanical ventilation in whom influenza is suspected, but not yet diagnosed, influenza testing should be performed on endotracheal aspirate specimens instead of those collected from the upper respiratory tract [61]. Molecular testing, including RT-PCR for influenza viruses can also be performed on bronchoalveolar lavage (BAL) fluid if collected for the testing of other pathogens. Blood, plasma, serum, cerebrospinal fluid, urine, and stool samples have very low diagnostic yield and are not recommended for influenza testing [49]. Diagnostic test results on specimens collected from non-respiratory sites should not be used for clinical decision making even for patients with extra-pulmonary complications of influenza.

Novel influenza A viruses are typically of animal origin, differ antigenically and genetically from currently circulating seasonal influenza A viruses (including H1N1pdm09 and H3N2 subtypes) and have infected at least one person. Novel influenza A viruses can cause a wide clinical spectrum of illness, ranging from asymptomatic infection, uncomplicated illness, to fulminant pneumonia, ARDS, and multi-organ failure [62] and human infection with a novel influenza A virus is of public health concern. In the U.S., human infection with a novel influenza A virus is nationally reportable to the Centers for Disease Control and Prevention; globally, under the International Health Regulations, countries are required to report such human cases to the World Health Organization. A major concern is the risk of novel influenza A virus transmission among humans; depending upon the prevalence of pre-existing immunity in the population, novel influenza A viruses may have pandemic potential. Patients suspected with novel influenza A virus infection should be investigated for a possible epidemiological link, i.e., a history of recent exposure to poultry or pigs or close contact to an individual with suspected or confirmed novel influenza A virus infection. Novel influenza A virus infection cannot be distinguished from seasonal influenza A virus infection by clinical findings or testing at clinical laboratories and therefore requires specific molecular testing of respiratory specimens by RT-PCR at public health laboratories [63]. Cases of suspected novel influenza A virus infections should be discussed with appropriate local and or national public health and laboratory staff to coordinate the testing of appropriate respiratory specimens.

## Treatment of influenza

Treatment of severe influenza presents multiple challenges. The mainstay of therapy for patients with influenza is initiation of antiviral medication as soon as possible after illness onset [49]. Currently available FDA-approved antiviral medications include neuraminidase inhibitors (NAIs) (e.g., oral oseltamivir, inhaled zanamivir, and intravenous peramivir); cap-dependent endonuclease inhibitor (baloxavir marboxil); and adamantanes (e.g., amantadine and rimantadine) (Table 4). NAIs and baloxavir have activity against both influenza A and B viruses. Adamantanes only have activity against influenza A viruses and are not recommended for treatment of influenza due to widespread resistance among currently circulating strains of seasonal influenza A viruses. Notably, FDA-approved antiviral medications for treatment of influenza are approved for early treatment of uncomplicated influenza in outpatients based upon randomized placebo-controlled clinical trials conducted among previously healthy outpatients. Meta-analyses of randomized placebo-controlled clinical trials of early oseltamivir treatment of influenza in pediatric and adult outpatients have reported clinical benefit in reducing duration of illness and risk for some complications associated with influenza [65, 66].

**Table 4:** Antiviral treatment

Antiviral agents and age group [64]	Treatment dosing
Oseltamivir	
Adults (including pregnancy)	75 mg twice daily
Children (1 year or older) ≤15 kg	30 mg twice daily
Children > 15–23 kg	45 mg twice daily
Children > 23–40 kg	60 mg twice daily
Children > 40 kg	75 mg twice daily
Term Infants 0–11 months*	*See details in footnote*
Preterm infants**	*See details in footnote*
Zanamivir	
Adults	10 mg (two 5-mg inhalations), twice daily
Children (≥ 7 years)	10 mg (two 5-mg inhalations), twice daily
Peramivir	
Adults	600 mg intravenous infusion once, given over 15–30 min
Children (2–12 years)	One 12 mg/kg dose, up to 600 mg maximum, intravenous, given over 15–30 min
Children (13–17 years)	600 mg intravenous infusion once, given over 15–30 min
Baloxavir marboxil***	
Adults and children (12 years or older) ≥40–80 kg	Single dose of 40 mg
Adults and children (12 years or older) ≥80 kg	Single dose of 80 mg

*FDA-approved oral oseltamivir treatment dose for infants 14 days and older and less than 1 year old is 3 mg/kg per dose twice daily. The American Academy of Pediatrics has recommended an oseltamivir treatment dose of 3.5 mg/kg orally twice daily for infants 9–11 months of age

**Current weight-based dosing recommendations are not appropriate for premature infants. Please refer to American Academy of Pediatrics recommendations (https://pediatrics.aappublications.org/content/142/4/e20182367) for further information

***Safety and efficacy of baloxavir marboxil in patients less than 12 years old or weighing less than 40 kg have not been established. There are no data on balosavir treatment of hospitalized patients with influenza, and appropriate dosing frequency is unknown. A phase III clinical trial of baloxavir treatment of hospitalized influenza patients is ongoing: https://clinicaltrials.gov/ct2/show/NCT03684044

No completed randomized, placebo-controlled trials of antiviral treatment have been conducted in hospitalized influenza patients to establish the efficacy of oseltamivir or other NAIs. A number of observational studies have reported clinical benefit of neuraminidase inhibitors in hospitalized patients, including reduction in duration of hospitalization and risk of death, including in ICU patients [67–74]. Additionally, a systematic review of published reviews/meta-analyses reported survival benefit of NAI treatment in hospitalized patients [75], although another meta-analysis of observational studies did not [69]. In particular, a large pooled individual patient-level meta-analysis of observational studies from 38 countries identified a 38% reduction in risk of mortality in critically ill adults and those aged ≥ 16 years old when comparing early NAI treatment (< 48 h) with later treatment (> 48 h), and a 69% reduction in mortality risk between influenza patients receiving early NAI treatment and those who did not receive NAIs [72]. The mortality risk reduction of NAI treatment at any time versus no treatment was 28% for critically ill patients aged ≥ 16 years old; while a similar reduction in mortality was identified in critically ill children aged < 16 years, the result was not statistically significant [72] and was likely underpowered because death is less common in hospitalized children with influenza than in adults.

Although studies have shown the greatest clinical benefit when antivirals are started within 2 days of illness onset, some observational studies have shown clinical benefit of neuraminidase inhibitors when started up to 5 days following symptom onset [15, 55, 76, 77]. The large meta-analysis mentioned above also identified a significantly reduced mortality risk reduction (35%) in critically ill patients aged ≥ 16 years old who received NAI treatment > 48 h after symptom onset compared with those who did not [72]. A cohort study of early versus late oseltamivir treatment reported a significant reduction in mortality and median duration of ICU hospitalization in severely ill patients with influenza A(H3N2), but not A(H1N1pdm09) or B virus infection in Greece [78]. One French study reported delays in initiation of oseltamivir treatment prescribed to hospitalized influenza patients and suggested empiric administration of oseltamivir treatment in the emergency department for patients being admitted with lower respiratory tract disease during influenza season [79]. Overall, based upon available observational data to date in hospitalized patients with influenza, including ICU patients, initiation of neuraminidase inhibitor antiviral treatment is recommended as soon as possible for hospitalized patients with suspected or confirmed influenza.

Data on optimal dosing and duration of therapy with neuraminidase inhibitors are limited in critically ill influenza patients. Enterically administered oseltamivir is the preferred treatment for most hospitalized patients, given the lack of data for intravenous peramivir in this population. The use of inhaled zanamivir is not recommended in in critically ill patients due to the lack of data in hospitalized patients and the risk of bronchospasm in patients with underlying lung disease. Studies indicate that oseltamivir administered orally or via oro/naso-gastric tube is well absorbed in critically ill patients and reaches plasma levels comparable to those in ambulatory patients [80]. Similarly, several observational studies indicate that enteric oseltamivir reaches comparable plasma concentrations to non-critically ill patients in those receiving extracorporeal membrane oxygenation (ECMO) and renal replacement therapy [80–87], although dosing should be reduced in patients with significant renal impairment. There is scant evidence that increased NAI dosing (e.g., twice daily dosing) in critically ill patients provides additional clinical benefit than standard dosing [80, 88–92]. Of note, studies also suggest that increased oseltamivir dosing does not provide additional clinical benefit in obese adults, including extreme obesity (BMI > 40) [93, 94]. Duration of therapy can be difficult to define, as prolonged influenza viral replication and shedding from the both upper and lower respiratory tract can occur in critically ill patients [95, 96]. For this reason, it may be beneficial to continue antiviral therapy beyond 5 days, and repeat virologic testing may be beneficial in determining appropriate therapeutic endpoints [97]. Continuing antiviral treatment in critically ill patients until virus is not detectable in the lower respiratory tract may also help reduce the pro-inflammatory dysregulated cytokine response triggered by influenza virus infection and reduce nosocomial influenza virus transmission to healthcare personnel in the ICU. Consultation with a specialist with training in infectious diseases for the potential emergence of antiviral resistant virus infection should be considered for ICU patients with evidence of persistent influenza viral replication after NAI treatment, particularly in severely immunocompromised patients [49, 98].

For patients who cannot tolerate or absorb enteric oseltamivir due to gastric stasis, malabsorption, or other gastrointestinal processes, intravenous peramivir may be an alternative [99, 100]; however, studies have not identified an advantage for intravenous peramivir in comparison with enteric oseltamivir [101]. Notably, a randomized trial conducted in three influenza seasons found similar clinical outcomes between IV peramivir and enteric oseltamivir in hospitalized adult influenza patients [102]; a separate trial did not identify significant additional clinical benefit of peramivir in combination with standard-of-care therapy (which often included an NAI) [103]. A more recent, multicenter randomized controlled trial also found similar clinical benefit between enteric oseltamivir and intravenous peramivir in hospitalized influenza patients [104].

In 2018, a novel antiviral agent, baloxavir marboxil, was FDA-approved for early treatment of uncomplicated influenza in outpatients aged ≥ 12 years old. Baloxavir acts via inhibition of the influenza virus cap-dependent endonuclease, a different mechanism than the neuraminidase inhibitors, and can treat NAI-resistant influenza virus infections. Randomized controlled trials of single-dose oral baloxavir showed similar clinical benefit to 5 days of twice-daily oral oseltamivir [105]. However, because these studies were limited to patients with uncomplicated influenza, the role of baloxavir monotherapy or in combination with an NAI for treatment of hospitalized influenza patients is unclear. Specifically, optimal dosing, duration of therapy, and appropriate endpoints have yet to be determined for baloxavir treatment of hospitalized influenza patients. In the outpatient RCT, patients treated with single-dose baloxavir showed significant reduction in influenza viral levels in the upper respiratory tract at 24 h compared with those receiving placebo or oral oseltamivir [105]. However, it is unknown whether this reduction in influenza viral shedding correlates with reduced transmissibility. A potential concern for the use of baloxavir in critically ill patients is the rapid development of resistance observed during the outpatient clinical trials [106]. A trial to assess the efficacy and safety of baloxavir in combination with oseltamivir versus oseltamivir monotherapy in hospitalized influenza patients is currently enrolling participants [107].

There are no completed randomized clinical trials of adjunctive corticosteroid treatment in influenza patients. A trial of corticosteroid therapy was planned during the 2009 H1N1 pandemic, but was halted due to limited number of enrolees [108]. One observational study in China during the 2009 H1N1 pandemic reported that administration of parenteral glucocorticoids within 72 h of illness onset tripled the risk of developing critical illness or death from influenza A(H1N1)pdm09 virus infection [109]. A re-analysis of prospectively collected data on 1846 influenza patients admitted with primary influenza pneumonia to 148 ICUs in Spain during 2009–2014 using propensity scoring matching reported that corticosteroid use was significantly associated with ICU mortality [110]. Meta-analyses of observational studies have concluded that that corticosteroid treatment of hospitalized influenza patients does not result in better outcomes and may be associated with adverse outcomes including increased mortality [111–113]. Similarly, a retrospective observational study conducted on critically ill children during the 2009 H1N1 pandemic found that high-dose (equivalent to 2 mg/kg per day of methylprednisolone) corticosteroid treatment was associated with mortality in the ICU, although a causative relationship was not determined [30]. A selection of individual observational studies in critically ill children and adults have also reported potential association between corticosteroid treatment and adverse influenza outcomes [30, 114, 115]. A recent Cochrane review of available observational studies suggested increased mortality when adjunctive corticosteroid therapy is used for influenza patients; however, the available evidence was of low quality and the authors suggest interpreting these results with caution [116].

Multiple studies have reported that corticosteroid treatment is associated with prolonged influenza viral shedding in hospitalized patients [117–119], including in sporadic human infections with avian influenza A(H7N9) virus in China [120], and increased rates of secondary bacterial and fungal co-infections [121, 122], which may lead to adverse clinical outcomes. However, there is some evidence to suggest that the increased risk attributed to corticosteroid treatment is a result of bias in observational studies. A large, retrospective study of critically ill adults in Canada found an increased risk of mortality in patients receiving corticosteroids; however, after adjusting for time-dependent differences between groups, no significant differences in mortality were observed with corticosteroid treatment [123]. Moreover, potential differences between low-dose and medium-/high-dose corticosteroid treatment are not well understood. One observational study of hospitalized patients with viral pneumonia due to avian influenza A(H7N9) virus infection in China reported that high-dose, but not low or moderate-dose corticosteroids, was associated with increased 30-day and 60-day mortality [124]. Currently, on the basis of available observational data to date, adjunctive corticosteroid treatment is not recommended for children or adults hospitalized with influenza, including critically ill patients, unless clinically indicated for another reason, such as treatment of asthma or COPD exacerbation or septic shock [49]. Further studies are required to understand the clinical benefit or harms associated with corticosteroid treatment of critically ill influenza patients.

Although neuraminidase inhibitors (oseltamivir) are currently recommended for antiviral treatment of influenza in hospitalized patients based on observational studies, including in critically ill patients, there are a number of novel strategies and products for treating influenza in various stages of development. One approach under investigation is triple-combination antiviral drug (TCAD) therapy, which combines amantadine, ribavirin, and oseltamivir for treatment of influenza in critically ill and high-risk patients. Unfortunately, studies to date have not shown a benefit of TCAD over oseltamivir monotherapy [125–127]. Several novel antiviral compounds are in various stages of investigation, including small-molecule polymerase inhibitors such as pimodivir [128] and favipiravir [129]. A number of monoclonal and polyclonal antibodies, targeted against a variety of influenza viral proteins, are also in development [130–133]. Similarly, convalescent plasma has shown potential benefit in the treatment of severe influenza, and further trials are underway [134–136]. Another area of intense interest is the modification of the host antiviral response to influenza virus infection. There are ongoing preclinical and clinical studies of a variety of other immunomodulatory agents for treatment of influenza, including celecoxib [137], statins, etanercept, pioglitazone, azithromycin [138], and interferons [139].

## Conclusions

Influenza vaccination can reduce the risk of complications from influenza, including reducing illness severity and the risks of hospitalization, ICU admission, and death. The elderly, young children, pregnant women, and those with underlying medical conditions are most at risk for severe complications of influenza. A diagnosis of influenza should be considered in critically ill patients admitted with complications such as exacerbation of underlying chronic comorbidities, community-acquired pneumonia, and respiratory failure during influenza season. Influenza molecular assays are recommended for testing upper respiratory tract specimens in patients without signs of lower respiratory tract disease. However, because critically ill patients with lower respiratory tract disease may have cleared influenza virus in the upper respiratory tract, but have prolonged influenza viral replication in the lower respiratory tract, an endotracheal aspirate (preferentially) or bronchoalveolar lavage fluid specimen (if collected for other diagnostic purposes) should be tested by molecular assay. Antiviral treatment with standard-dose oseltamivir delivered orally or enterally by oro or naso-gastric tube is recommended as soon as possible for patients with suspected influenza without waiting for testing results. Corticosteroids should not be routinely administered for treatment of influenza and should only be given for other indications (e.g., exacerbation of asthma or chronic obstructive pulmonary disease, or septic shock), because of the risk for prolongation of influenza viral shedding and ventilator-associated pneumonia in critically ill influenza patients with respiratory failure. Future directions for treatment of influenza in critically ill patients include novel antiviral compounds, combination antiviral treatment with drugs with different mechanisms of action, immunomodulatory agents, and strategies for multi-modality, combination antiviral, and host-directed immunomodulatory therapies.

## Footnotes

1 These risk factors are included in the U.S. CDC’s Advisory Committee on Immunization Practices recommendations for influenza vaccination. This may also apply to indigenous people from other countries, including indigenous Australians and First Nations people.
